# Automated Biosurveillance Data from England and Wales, 1991–2011

**DOI:** 10.3201/eid1901.120493

**Published:** 2013-01

**Authors:** Doyo G. Enki, Angela Noufaily, Paul H. Garthwaite, Nick J. Andrews, André Charlett, Chris Lane, C. Paddy Farrington

**Affiliations:** Author affiliations: Open University, Milton Keynes, UK (D.G. Enki, A. Noufaily, P.H. Garthwaite, C.P. Farrington);; Health Protection Agency, London, UK (N.J. Andrews, A. Charlett, C. Lane)

**Keywords:** algorithms, biosurveillance, data analysis, database, epidemiology, infectious disease outbreaks, public health surveillance, statistical model, England, Wales

## Abstract

Outbreak detection systems for use with very large multiple surveillance databases must be suited both to the data available and to the requirements of full automation. To inform the development of more effective outbreak detection algorithms, we analyzed 20 years of data (1991–2011) from a large laboratory surveillance database used for outbreak detection in England and Wales. The data relate to 3,303 distinct types of infectious pathogens, with a frequency range spanning 6 orders of magnitude. Several hundred organism types were reported each week. We describe the diversity of seasonal patterns, trends, artifacts, and extra-Poisson variability to which an effective multiple laboratory-based outbreak detection system must adjust. We provide empirical information to guide the selection of simple statistical models for automated surveillance of multiple organisms, in the light of the key requirements of such outbreak detection systems, namely, robustness, flexibility, and sensitivity.

## Abstract 2

Twenty years of data provide valuable insights for the design of large automated outbreak detection systems.

The past decade has witnessed much interest in real-time outbreak detection methods for infectious diseases, driven by worries about the possibility of large-scale bioterrorism, public concern about emerging and reemerging infections, and the increased availability of computerized data (1–3). More prosaically, outbreaks of commonly occurring pathogens, notably, those causing infectious intestinal disease, remain a serious public health issue, causing an appreciable number of deaths and imposing a substantial drain on public health resources in many countries (4,5).

In England and Wales, automated laboratory surveillance of infectious diseases has been undertaken since the early 1990s. Laboratory surveillance is based on counts of laboratory isolates of infectious pathogens, usually classified for epidemiologic purposes by subtype or phage type. The organism reports come mainly from samples sent to hospital laboratories or to specialist laboratories when additional typing is required, as for salmonellae.

This automated system was designed to supplement the frontline investigator-led outbreak detection methods used by national and regional epidemiologists, with the primary aim of identifying geographically distributed outbreaks that may have escaped local detection. In a typical week, several hundred different pathogens are reported; the automated system provides a back-up and the assurance that the entire database is routinely scanned. The output comprises a short list of organisms with potential outbreaks for review, ranked according to an exceedance score that measures the degree of statistical aberrance. The statistical methodology of the system was described previously (6) and has since been applied in Scotland (7) and in several other European countries (8).

Much research on statistical methods of prospective outbreak detection has been aimed at identifying unusual clusters of 1 syndrome or disease (9–12), and some work has focused on multivariate surveillance methods (12). However, little research has been directed toward developing outbreak detection methods that are suited to large, multiple surveillance systems involving thousands of different organisms, such as the system used in England and Wales.

We are reviewing the statistical methods used in the England and Wales system. The first stage of this review, reported here, has been to carry out a detailed analysis of the data accumulated over the 2 decades since 1991. We aimed to document some of the generic features of surveillance data and their imperfections across the range of organisms of interest and to identify the key problems confronting automated outbreak detection systems. Specifically, we endeavored to answer 2 key questions: How diverse are the patterns displayed by the range of organisms monitored? How complex must a statistical algorithm be to handle this diversity?

## Data and Methods

### Data

The data were provided by the Health Protection Agency (www.hpa.org.uk) from their LabBase surveillance database. This is a computerized database that receives details of all organisms reported by participating laboratories (the numbers of which vary from week to week) in England and Wales. The data are routinely subjected to intensive de-duplication checks at the time of report. A single report contains a data trail that starts with the date of collection of the first specimen and ends with the date at which the complete identification of the organism is entered into the database. The delay between the first specimen date (hereafter referred to as specimen date) and the date of final entry (referred to as report date) is a key feature of all systems of laboratory surveillance. The outbreak detection system operates on the basis of report dates (current and historical); the alternative is to operate on the basis of specimen dates, which requires explicit modeling of the delay distribution.

The outbreak detection system runs automatically every weekend, processing the previous week’s reports. Thus, the time unit of analysis is by the week unless otherwise specified. We obtained weekly counts of all infectious disease organisms reported to the Health Protection Agency between week 1, 1991, and week 52, 2011, by date of report and date of specimen collection. In years with 53 weeks, the week 53 count was added to the week 1 count of the following year. To mitigate the effect of delays at the end of the series, only isolates with specimen dates through week 26 of 2011 were used in the analyses. All analyses are by week of specimen collection unless otherwise specified.

### Data Processing

Calculating rates and other organism-specific statistics is complicated by the fact that it is not possible to distinguish between genuine zeroes, corresponding to organisms looked for but not found, and missing values that arise when organisms are not sought. It is highly likely that some organisms that were identified toward the end of the study period would not have been identified by the tests that were performed a decade or so earlier. Rates and trends calculated without taking any account of this feature would be biased. To reduce this bias, we recoded all leading sequences of zeroes as missing. However, this in turn introduces a selection bias, because every time series would then start with a nonzero count. To mitigate this, we reduced the first nonzero count by 1.

### Statistical Models

The statistical models are described informally; a technical account is provided in the Technical Appendix. To summarize the mean frequencies, trends, and seasonality of each organism, we used log-linear models structured as follows:

log (average count in week *t*) = baseline + trend at week *t* + seasonality at week *t*

We fitted a range of such log-linear models to the data, incorporating a smooth long-term trend component and monthly seasonality for each series of organism counts (13,14). A key aim was to identify a simple family of models that adequately represents all organisms. The simplest model is the Poisson model, for which the variance of the count in week *t* equals its average value. The model is convenient and easy to automate, but restrictive. We therefore considered other convenient but less restrictive models where

Variance of count in week *t =* dispersion × average count in week *t* (1)

Models of this form are called quasi-Poisson. The dispersion in Equation 1 is a constant specific to each organism. In a Poisson model, the dispersion is equal to 1. When the dispersion is >1, more variability is thereby allowed.

We also investigated the negative binomial model. This model satisfies equation 1 but also allows a greater degree of skewness (that is, asymmetry around the mean) than the Poisson model. For the negative binomial model, 

Skewness of count in week *t =* antilog [constant – 0.5 × log (average count in week *t*)] (2)

where the constant in equation 2 is nonnegative. For the Poisson model, this constant is zero: equation 2 allows greater positive skewness.

### Model Evaluation: Relationships between Mean, Variance, and Skewness

We sought a simple family of models that adequately describes all organisms, rather than a well-fitting model for any particular organism. Formal goodness-of-fit tests were not used because they can be unreliable with sparse data. Our criterion was that the relationships between mean, variance, and skewness should be adequately described. To display these relationships for each organism, we subdivided the data into 41 half-years, dropped weeks 52 (or 53) and 1 (which are atypical, as noted above), and de-seasonalized the data. We then calculated the mean, variance, and skewness in each half-year.

For each organism, we investigated the validity of equation 1 by plotting the log of the variance of the weekly counts against the log of the average weekly count in the 41 half-years. If equation 1 holds, the points should lie on a straight line with slope 1. We obtained the histogram of these slopes; a narrow spread around 1 suggests that the quasi-Poisson model is adequate.

Similarly, we investigated the validity of equation 2 by plotting the skewness of the weekly counts against the log of the average weekly counts. If equation 2 holds, the points should lie on the curve determined by this equation, for which the coefficient of the log of the average weekly count is −0.5. We obtained these coefficients and plotted their histogram; a narrow spread around −0.5 suggests that the negative binomial model is adequate.

## Results

We present the results in 5 subsections: global features of the surveillance system; frequency distributions; means, seasonality and trends; dispersion; and relationships between mean, variance, and skewness. Additional details are available in the online Technical Appendix.

### Global Features of the Surveillance System

**Figure 1:**
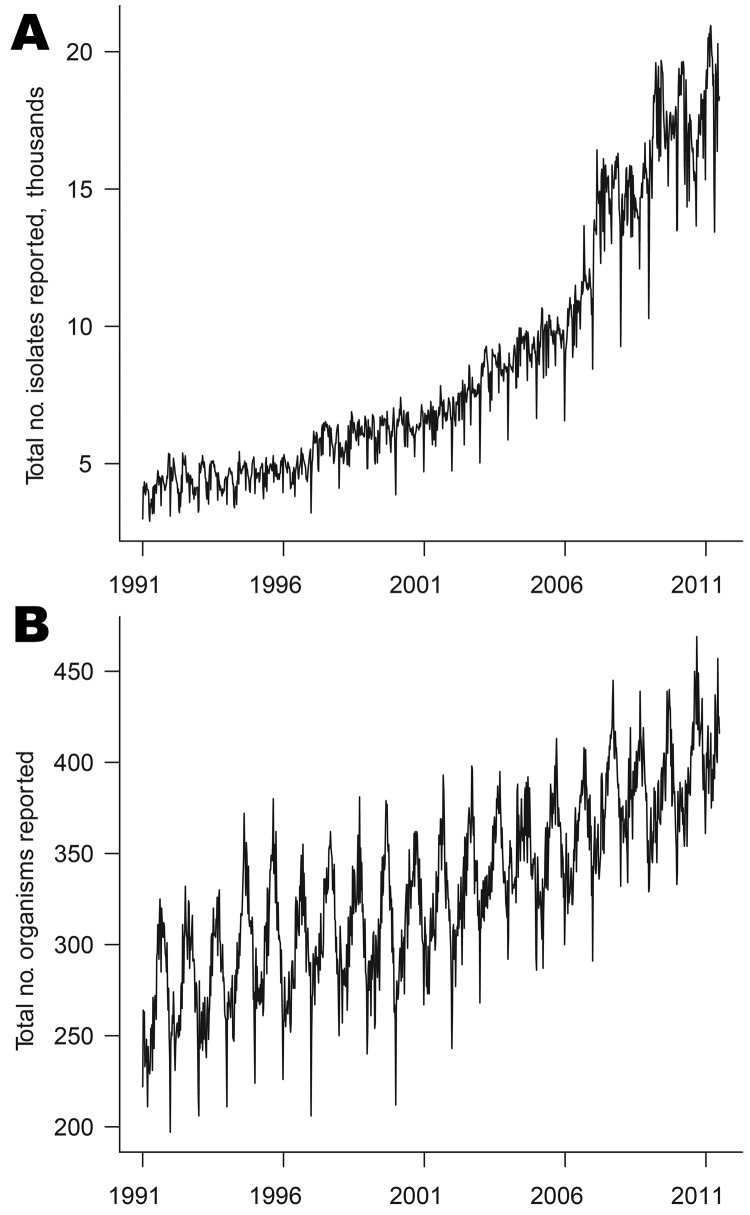
Weekly counts of organisms by date of specimen collection, England and Wales, 1991–2011: A) isolates; B) organism types.

More than 9 million individual isolates were collected with specimen dates from week 1 in 1991 through week 26 in 2011. These isolates were of 3,303 different organism types. Figure 1 shows the time series of counts of organism types and organism isolates by week of specimen collection. The number of types is highly seasonal with summer peaks; such seasonality is less apparent for individual isolates because of the large number of distinct, rare enteric infections. Also apparent are troughs at weeks 52 and 1, representing lower activity over the Christmas period.

The strong upward trends shown in Figure 1 represent a genuine increase in numbers of isolates and organism types over time, rather than an increase in the number of reporting laboratories. The numbers of laboratories reporting to the system tended to decline over time (Technical Appendix Figure 1). When ordered by date of report, the number of laboratories reporting is fewer than when ordered by date of specimen, reflecting batching of reports (some laboratories wait to accumulate isolates before reporting them). This factor is a notable source of additional noise in the surveillance system when considering counts by week of report. This batching will also affect the timeliness of the surveillance system.

On average, the weekly count of isolates is the same, whether ordered by week of specimen or by week of report; this is also true of organism counts. The variation in the differences in counts reflects the variability in delays from specimen collection to reporting, which can be considerable (Technical Appendix Figure 2).

The distribution of delays between date of specimen and date of report varies from organism to organism, with the median typically in the range of 7–28 days, depending on the complexity of the laboratory procedures involved. For example, modal delays for salmonellae are increased by the additional subtyping step required. Extreme delays are not uncommon, owing to late submissions or data entry errors (Technical Appendix Figure 3).

### Frequency Distributions

**Figure 2:**
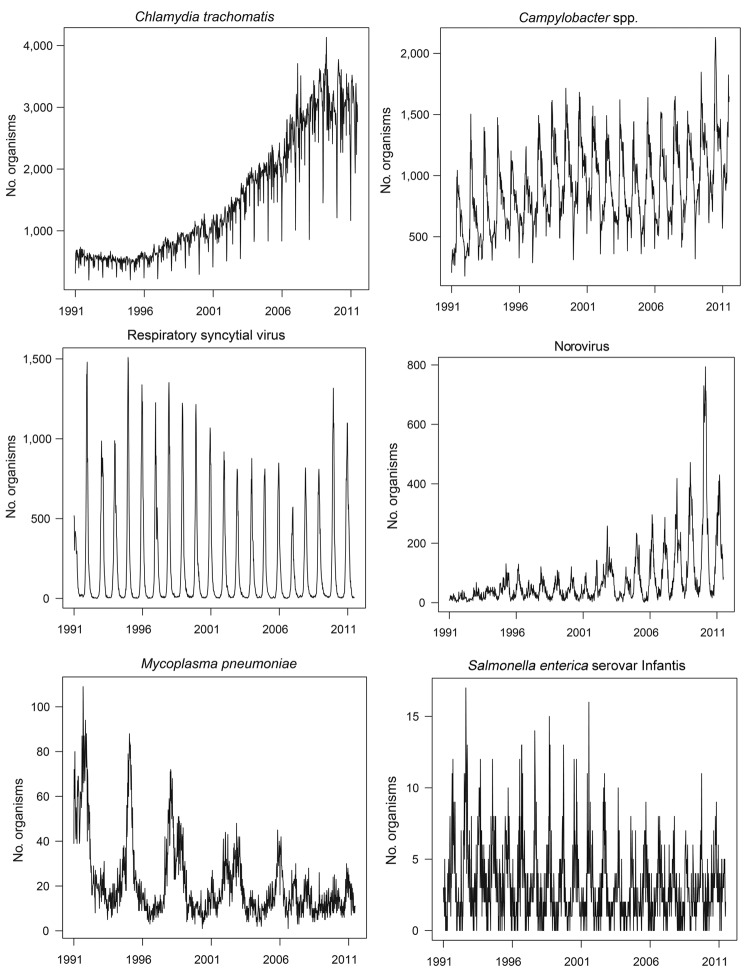
Weekly counts for 6 selected organisms, by date of specimen collection, England and Wales, 1991–2011.

There is huge variation in frequency, seasonality, and trends among the 3,303 organism types reported. Figure 2 exemplifies this variation, even among more common organisms. Most organisms were seldom reported; of the 3,303 recorded organisms, nonzero counts occurred in only 1 week for 637 organisms (19%), in only 2 weeks for 291 (8.8%), and in only 3 weeks for 225 (6.8%). At the other end of the scale, for 30 organisms the count by week of specimen was nonzero for each of the 1,070 weeks (including the 4 occurrences of a 53rd week) spanning the period. These organisms are listed in Table, along with the mean weekly counts by week of specimen.

**Table:** Organisms (mean weekly count, by specimen collection week) with nonzero specimen counts in every week from 1991 to mid-2011, England and Wales

Organism name	Mean weekly count
*Chlamydia trachomatis*	1,480
*Campylobacter* spp.	899
*Staphylococcus aureus*	764
Clostridium difficile toxin detection	313
Rotavirus	303
*Escherichia coli* untyped	267
*Staphylococcus coagulase* negative	167
*Streptococcus pneumoniae*	119
Herpes simplex virus untyped	102
Herpes simplex virus type 2	100
*Pseudomonas aeruginosa*	96
Herpes simplex virus type 1	92
*Cryptosporidium* spp.	86
*Giardia lamblia*	86
*Clostridium difficile* not stated	81
Norovirus	76
*Streptococcus* group B	57
*Mycobacterium tuberculosis*	54
*Enteroccoccus faecalis*	52
*Klebsiella pneumoniae*	50
*Streptococcus* group A	49
Adenovirus untyped	43
*Staphylococcus epidermidis*	36
*Proteus mirabilis*	35
*Enterobacter cloacae*	34
Cytomegalovirus	29
*Streptococcus* group G	26
*M. pneumoniae*	21
*Enterococcus faecium*	19
*Bacteroides fragilis*	14

This variation in the number of nonzero counts is mirrored by the maximum weekly count for each organism. For 90% of all organisms, the weekly maximum was <12; 1,651 (50%) had a maximum weekly count of 1. The remaining 10% includes several organisms with maximum weekly counts of several thousand, such as *Chlamydia trachomatis* (maximum 4,133) and *Staphylococcus aureus* (maximum 2,317).

### Means, Seasonality, and Trends

**Figure 3:**
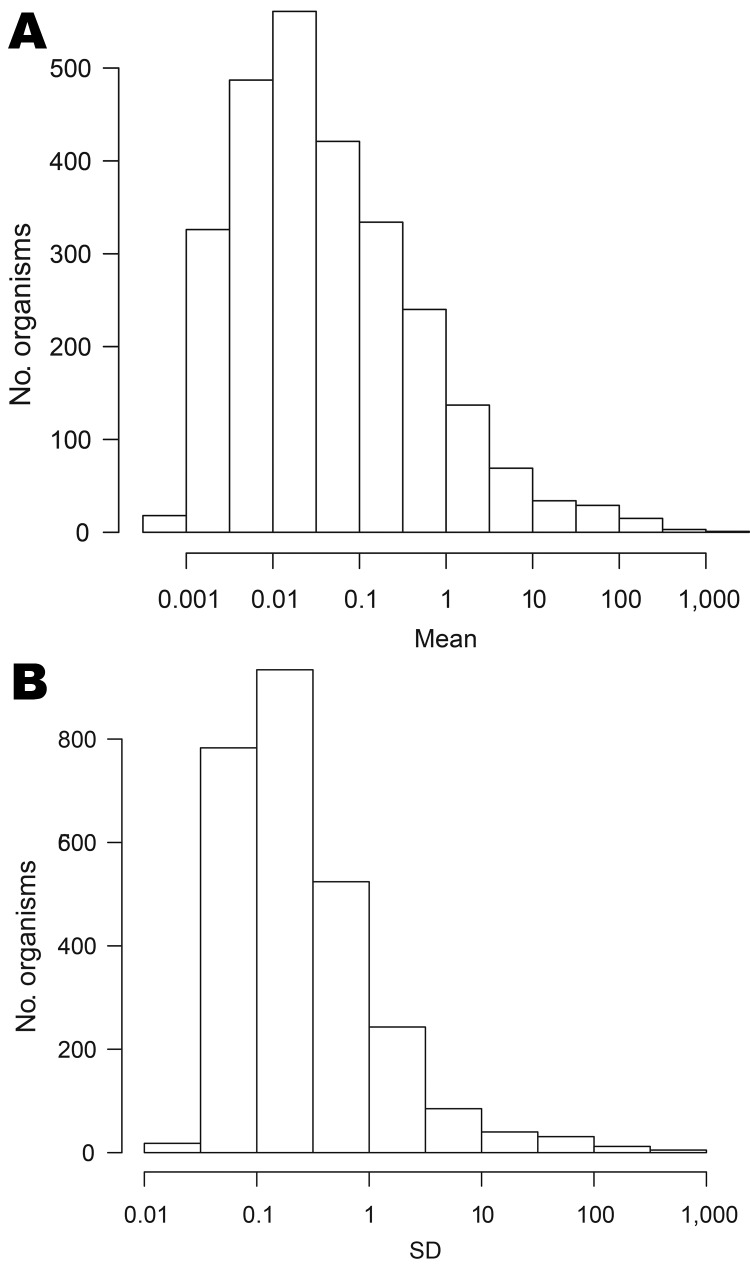
Distributions of mean (A) and SD (B) of weekly counts for all organisms, England and Wales, 1991–2011.

The large increase in numbers of organisms reported over time (Figure 1, panel B) suggests that laboratory procedures have changed over time. A total of 2,675 organisms with nonzero counts were left after recoding leading sequences of zeroes. The median weekly count for 2,408 (90%) of these organisms was zero. Figure 3 shows histograms of the mean and standard deviation of weekly counts by organism. The means span 6 orders of magnitude. There is also much variation in standard deviations, the 3 largest being for *C. trachomatis* (982.6), *S. aureus* (637.8), and rotavirus (338.4). The means for these organisms are 1,480, 764, and 303, respectively.

**Figure 4:**
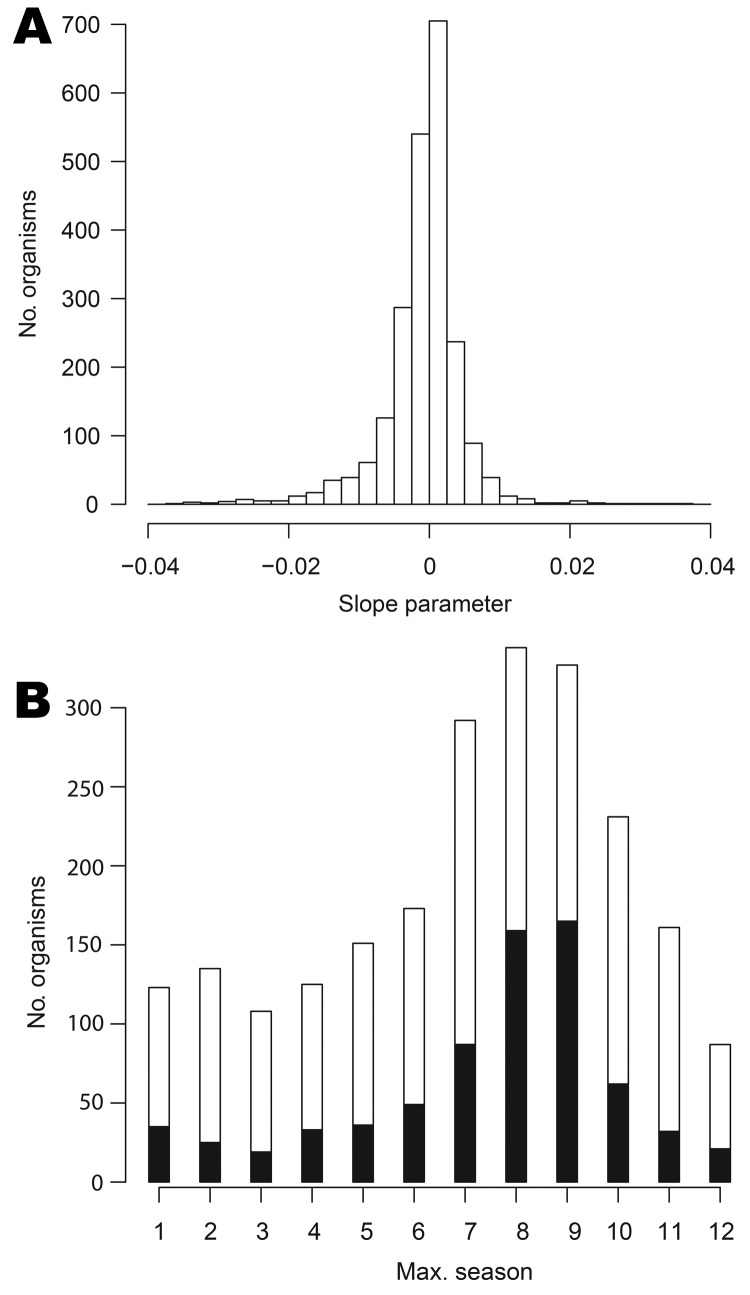
A) Distribution of estimated linear trend parameters (units: per week) for data on 2,250 organisms (excluding 4 organisms with extreme slopes), England and Wales, 1991–2011. B) Stacked bar chart of modal seasonal period for 2,254 organisms. The black bar sections represent organisms for which the seasonal effect is statistically significant.

We fitted log-linear models to the 2,254 organisms for which nonzero counts spanned >1 year. The distribution of slope parameters for linear trend is shown in Figure 4, panel A: 1,107 organisms display some evidence of an increasing trend (positive slopes, of which 655 are significant at the 5% level) and 1,146 of decreasing trend (negative slopes, 683 are significant).

Figure 4, panel B, shows the bar chart of the modal season for the 2,254 organisms analyzed. Every period is modal for some organism, though organisms with summer peaks predominate. However, the seasonal effect was significant at the 5% level for only 723 (32%) of the 2,254 organisms analyzed.

Some organisms displayed evidence of nonconstant seasonality. Rotavirus, for example, which typically peaks in the early months of the year, had slightly earlier peaks in the earlier years of data collection.

### Dispersion

For 1,333 (59%) organisms, the dispersion (that is, the ratio of variance to mean, equation 1) is >1, indicating that the variability of weekly counts of that organism is greater than that of a Poisson distribution. There is a general tendency for the dispersion to increase with the mean: the more common the organism, the less appropriate a Poisson model tends to be (Technical Appendix Figure 4, left panel). For many organisms, the dispersion is greater when calculated from data based on week of report, than when calculated from data based on week of specimen (Technical Appendix Figure 4, right panel). This extra variability likely reflects the extra clustering induced by reporting delays. The increase in the dispersion primarily affects the more common organisms. The mean value of the ratio of the 2 dispersion values is 1.14 (median 1); when restricted to organisms for which the dispersion by specimen date is >1, it is 1.26 (median 1.04).

In some cases, a contributing factor to the extra variation is large systematic variation in diagnostic practice, resulting in large variations in reporting intensity, notably, long runs of zeroes, as with *Helicobacter pylori* (Technical Appendix Figure 5). However, such patterns appear to be unusual; it is most likely that the extra variation is caused by clustering of cases in time, which can be accommodated relatively simply by a suitable choice of statistical model, to be discussed next.

### Relationships between Mean, Variance, and Skewness

**Figure 5:**
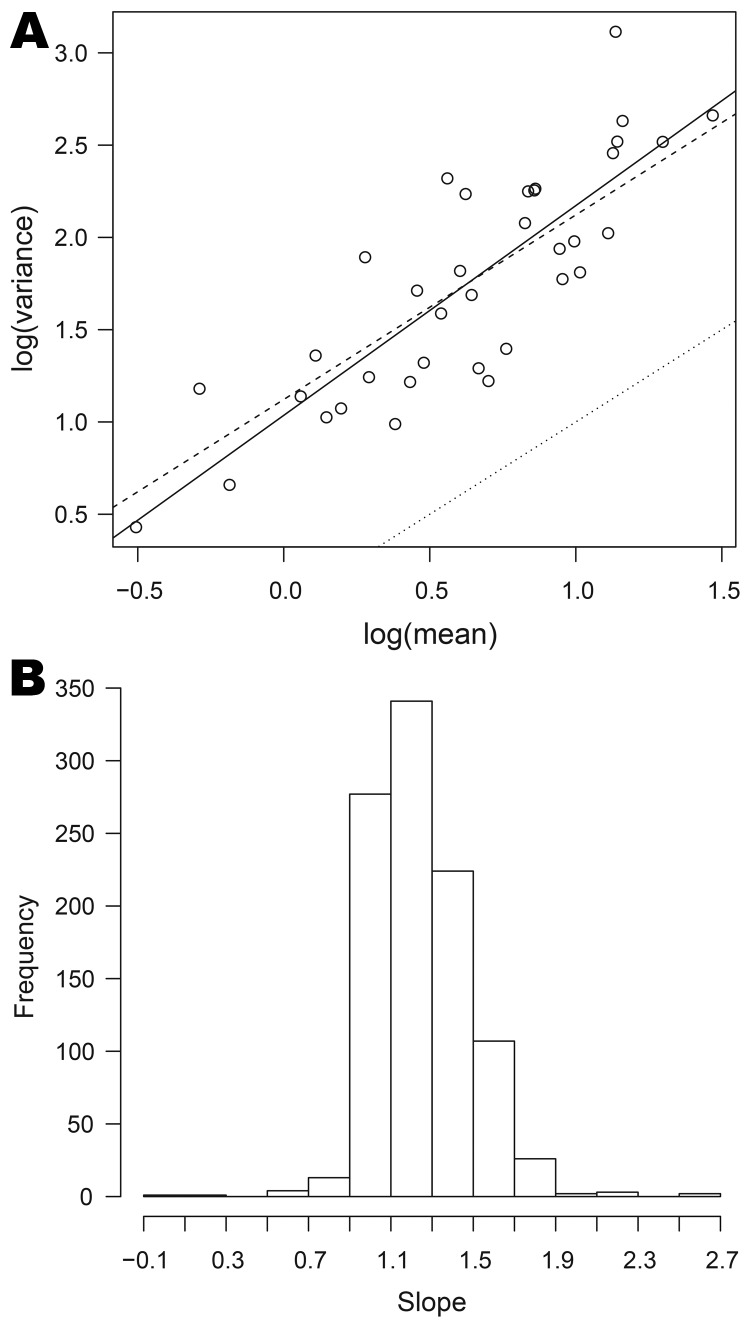
Relationships between mean and variance for data on organisms collected, England and Wales, 1991–2011. A) The log of variance plotted against log of mean for *Cyclospora* spp. The full line is the best fit to the points; the dashed line corresponds to the quasi-Poisson model; the dotted line corresponds to the Poisson model. B) Histogram of the slopes of the best-fit lines for 1,001 organisms; the value 1 corresponds to the quasi-Poisson model (equation 1).

Relationships between mean, variance, and skewness were investigated for the 1,001 organisms with dispersion >1 for which nonzero means and variance were obtained for >3 half-years. In all cases, the scatterplot of log(variance) against log(mean) was remarkably linear. Figure 5, panel A, shows this relationship for *Cyclospora* spp. (the full set of plots is available from the corresponding author). The full line is the best fit line through the points, and lies some way above the dotted line, which corresponds to the Poisson model. The dashed line corresponds to a quasi-Poisson model (equation 1). The closeness of the dashed line to the full line suggests that this model is not unreasonable for *Cyclospora* spp.

For 538 (54%) of these 1,001 organisms, the slope of the best-fit line is significantly different from 1, the value corresponding to the quasi-Poisson model, and in 535 of these the slope is >1 (the exceptions are *Providencia stuartii, Mycobacterium bovis* (bacillus Calmette-Guérin strain), and *Neisseria meningitidis* serotype B not further typed). This indicates that there is statistical evidence against the quasi-Poisson in about half the cases. However, departures from the quasi-Poisson model are typically moderate, the slope parameters lying for the most part between 0.9 and 1.7 (and thus reasonably close to 1), as shown in Figure 5, panel B.

**Figure 6:**
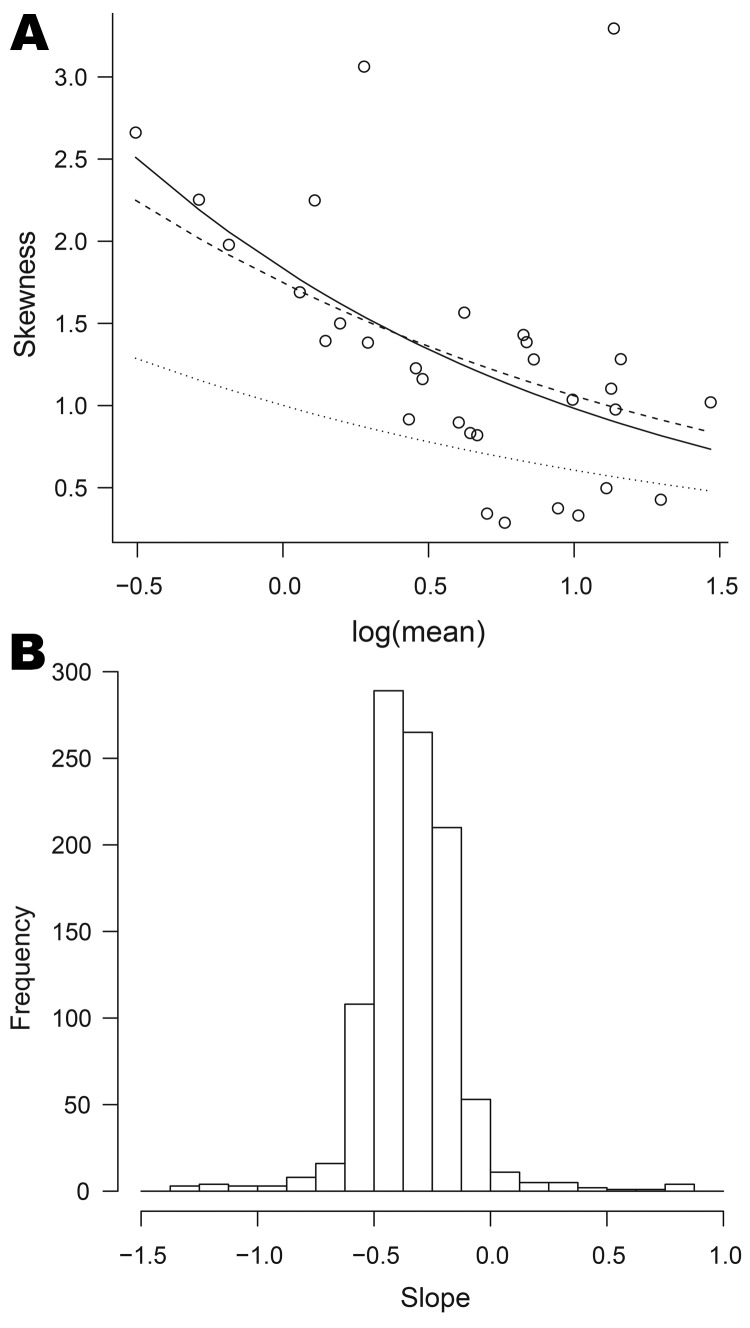
Relationships between mean and skewness for data on organisms collected, England and Wales, 1991–2011. A) Skewness plotted against log of mean for *Cyclospora* spp. The full curve is the best fit to the points; the dashed curve corresponds to the negative binomial model; the dotted curve corresponds to the Poisson model. B) Histogram of the parameters corresponding to the best-fit curves for 1,001 organisms; the value −0.5 corresponds to the negative binomial model (equation 2).

Most organisms, other than the most common, displayed a degree of positive skeweness, that is, long upper tails. The plots of skewness against log(mean), though often broadly exponential, showed more scatter than those of log(variance) against log(mean). Figure 6, panel A, shows the plot for *Cyclospora* spp., the dashed line now corresponding to the negative binomial model (the full set of plots is available from the corresponding author).

For 486 (49%) of the 1,001 organisms, the slope parameter (on the log scale) is significantly different from −0.5, the value corresponding to the negative binomial. For 475 of these it was greater than −0.5. Again, departures from this reference value were moderate, most slope parameters lying between −0.6 and 0, as shown in Figure 6, panel B.

What these results signify is that the quasi-Poisson model provides an adequate, though far from perfect, account of the week-to-week variability in organism counts for the broad range of organisms considered. The negative binomial model may also provide an adequate representation of these highly heterogeneous data; because this model accounts for the skewness in the data, which the quasi-Poisson model does not, it may provide more accurate threshold values, above which counts are declared to be aberrant.

## Conclusions 

We have undertaken a detailed analysis of the global features of a large surveillance database accumulated during >20 years. Most striking is the variety of temporal patterns, in terms of frequencies, trends, and seasonality. Some valuable general conclusions emerge of direct relevance to the design of outbreak detection systems.

The first stems from the great variation in organism frequency, which stretches over 6 orders of magnitude (from 10^−3^ to 10^3^ per week). The sensitivity and specificity of the detection system should remain broadly constant over this range, so that the system performs well for both rare and common organisms. The primary output from a multiple outbreak detection system is likely to be a ranking of aberrances in decreasing order of the statistical evidence underpinning them. The correctness of the ordering is arguably more important than achieving nominal sensitivity and specificity levels, so that attention is focused on the most discrepant organisms. In practice, this means that outbreak detection methods used with multiple surveillance systems must perform robustly and consistently over the range of frequencies expected (or a large part of this range).

A second conclusion is that the systematic components of the statistical outbreak detection models must be able to cope automatically with the idiosyncrasies of individual data series, notably seasonality and trends, without requiring intervention by the user. This necessitates the use of suitably flexible modeling environments, though excessive flexibility can itself cause problems of overfitting. A careful balance needs to be struck: for example, between the detailed modeling required to incorporate seasonal effects, which is crucial for some organisms, while recognizing that such effects are not greatly relevant for many others. In addition, robust numerical algorithms that are guaranteed to work for all but known extreme data configurations are essential.

Third, our analyses provide empirical support for the use of a single, robust algorithm across this range of organisms. The data suggest that the great majority of organisms can adequately—though far from perfectly—be represented by a statistical model in which the variance is proportional to the mean, such as the quasi-Poisson or negative binomial models. Some improvement would nevertheless be possible through the use of more general models in which the variance is proportional to a power of the mean. Such more general distributions, based on birth processes, have been studied (15); further investigation is warranted for application to surveillance data.

These conclusions apply specifically to the use of automated biosurveillance as a second line of defense in support of investigator-led outbreak detection methods, as implemented in England and Wales. Thus, we seek a system that performs adequately over the entire range of organisms, to be scanned routinely, rather than one that is optimized for a particular organism. We believe that integrating investigator-led and automated surveillance in this way plays best to the strengths of each method.

Each week, the England and Wales detection system flags ≈20 organisms, listed in decreasing order of aberrance, for further investigation. A proportion of these results are false positive and do not correspond to a genuine outbreak. The remainder are genuine outbreaks, many of which will also have been picked up by the front-line investigator-led network of surveillance specialists, as intended. Occasionally, genuine outbreaks are picked up which have escaped detection by other means. These events often involve pathogens with a wide geographic distribution and relatively high baseline frequency of reporting. Such dispersed outbreaks may be overlooked at the local level, where they often equate to only marginal increases, but nationally may represent noteworthy events. Recent examples include outbreaks of *Salmonella enterica* serotype Enteritidis phage type 14b in 2009 (16), *S. enterica* ser. Java in 2010 (17), *S. enterica* ser. Montevideo in 2011, and *S. enterica* ser. Poona in 2012.

Our current efforts at improving the system are to reduce the false-positive rate while maintaining sufficient power to detect genuine outbreaks. Some of the key issues to be revisited are treatment of trends, seasonality, and calculation of thresholds, in the light of the findings presented here. Other issues are how to handle past outbreaks and delays between specimen collection and reported identification. The data and experience gained from >20 years’ of automated biosurveillance will provide valuable empirical underpinning for such improvements.

Technical Appendix Automated biosurveillance data from England and Wales, 1991–2011. This online appendix provides technical details of statistical methods, further technical description of results, and 5 supplemental figures.
