# Supplementary material for: Automated Biosurveillance Data from England and Wales, 1991–2011
Source: Emerg Infect Dis. 2013 Jan;19(1):35–42. doi: 10.3201/eid1901.120493 (PMC3557985; doi:10.3201/eid1901.120493)
Supplement: Technical Appendix — Automated biosurveillance data from England and Wales, 1991–2011. This online appendix provides technical details of statistical methods, further technical description of results, and 5 supplemental figures. [file 12-0493-Techapp-s1.pdf]

Article DOI: http://dx.doi.org/10.3201/eid1901.120493

Automated Biosurveillance Data from England and Wales, 1991–2011

Technical Appendix

This online appendix provides technical details of statistical methods; further technical description of results; and online Technical Appendix Figures 1 to 5 referred to in the main text. References are numbered as in the main text.

Statistical Methods

The models used to analyse the data were quasi-Poisson models of the form

$E(y_t) = \mu_t$, $\mathrm{var}(y_t) = \phi\mu_t$ with $\phi \geq 1$ (A)

where y_t is the count of a particular organism in week t and ϕ is a dispersion parameter representing extra-Poisson variability when $\phi > 1$. Generalised linear models (GLM) of the form

$\log(\mu_t) = \alpha + \beta t + \mathrm{seas}(t)$ (B)

were used, where seas(t) is a 12-level factor representing seasons, the factor levels roughly corresponding to calendar months (*13*). In analyses where more detailed modelling of trends was required we used generalized additive models (GAM) of the form

$\log(\mu_t) = \alpha + s(t) + \mathrm{seas}(t)$ (C)

where $s(t)$ represents a smooth function of time (*14*). Time t was centred in all analyses.

Estimation of the regression parameters was by maximum quasi-likelihood, and inclusion of model terms was tested using the likelihood ratio test. The dispersion parameter was estimated by dividing the Pearson chi-square by the degrees of freedom (*13*, p. 200). To avoid overfitting the model with sparse data, if the estimated value of ϕ was less than 1 we forced it to equal 1 (ϕ was only ever less than 1 with sparse data).

A natural statistical model for surveillance data is the negative binomial model, describing a random variable which is conditionally Poisson with mean ν, with ν itself a random variable with a gamma density of mean μ. This is a negative binomial distribution

with mean μ and variance of the form $\phi\mu$, with $\phi > 1$ (*13*, p. 199). The skewness of the distribution is $(2\phi - 1) / (\phi\mu)^{1/2}$.

To study the mean-variance and mean-skewness relationships empirically we first de-seasonalized the data using the transformation

$z_t = y_t/\exp(\text{seas}(t))$, (D)

where y_t is the observed count and seas(t) is the fitted seasonal factor. Mean-variance relationships were analysed by plotting the log of the variance of z_t against the log of the mean of z_t in adjacent 6-month periods. The line log(var) = log(mean) corresponds to the Poisson distribution, and the line log(var) = log(ϕ) + log(mean) corresponds to the negative binomial model. Consistency of the data with the latter was investigated by testing the null hypothesis that the slope in the normal errors regression of log(var) against log(mean) is 1. Similarly, we plotted the skewness of z_t against the log of the mean of z_t . The curve skewness = exp(–0.5 log(mean)) corresponds to the Poisson distribution, and the line skewness = $\exp(\log(\phi^{-1/2} (2\phi - 1)) - 0.5 \log(\text{mean}))$ corresponds to the negative binomial model. Consistency of the data with the latter was investigated by testing the null hypothesis that the coefficient of log(mean) in the normal errors regression with log link is –0.5.

Formal goodness of fit tests were not employed, owing to the sparsity of the data for many organisms.

Results

Means, Seasonality and Trends

Linear trends were investigated using the model in equation B, with slope parameter β. We investigated seasonality as follows. When the estimated value of α in the GLM of equation A was non-negative, as was the case for 283 organisms, we fitted the GAM of equation B. We did not seek to fit the GAM to sparse data (defined as those organisms with $\alpha < 0$) owing to convergence problems. Seasonality was assessed from the final model fitted for each time series.

Dispersion

We studied overdispersion relative to the Poisson distribution for all 2,254 organisms. We first obtained the log of the mean weekly count, α. Then for organisms with specimen dates spanning 52 or fewer weeks we fitted the GLM of equation A and obtained the

dispersion parameter, ϕ. For organisms spanning more than 52 weeks we used the procedure described previously, fitting a GLM or a GAM according to the value of α, and obtained the dispersion parameter ϕ. The means shown in online Technical Appendix Figure 4 (left) and Figure 4 (right) are the values of exp(α) (the data were centred prior to analysis).

Relationships between Mean, Variance and Skewness

Figure 5 (B) and Figure 6 (B) of the main text show histograms of the slope parameters obtained for the two regressions, of log(variance) against log(mean), and of skewness against log(mean). The median value of the slope parameter for the linear regression of log(variance) on log(mean) was 1.2, corresponding to a variance function proportional to $\mu^{1.2}$. For the log-linear regression of skewness against log(mean), the median slope parameter was –0.34, corresponding to a skewness function proportional to $\mu^{-0.34}$. Thus, the data tend to exhibit greater variance and skewness than under the negative binomial model, though very substantial departures from it are uncommon.

The quasi-Poisson and negative binomial models provide reasonable compromises if a single model is sought for all organisms, though there is clearly room for improvement, perhaps by allowing a more general power dependence between the variance (and the skewness) and the mean.

Technical Appendix Figures 1 to 5

All additional figures are referred to in the main text. Technical Appendix Figure 4 is also referred to under Results.

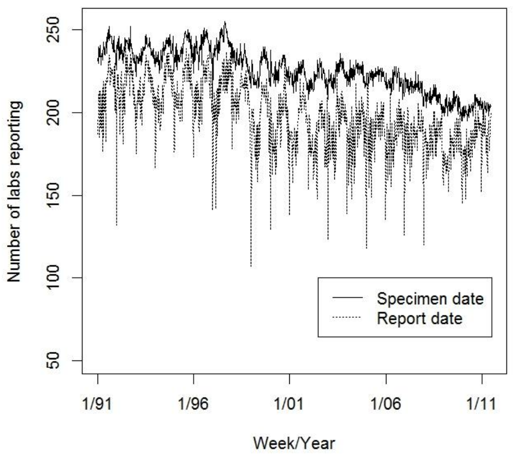

Technical Appendix Figure 1. Number of laboratories reporting by date of specimen and date of report. Both decline over time. The numbers by date of report are lower than the number by date of specimen, suggesting batching of reports.

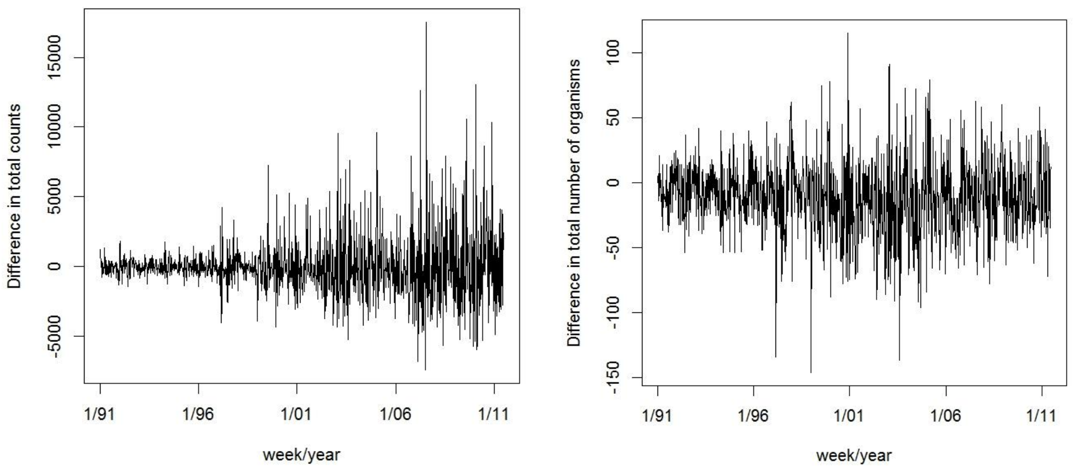

Technical Appendix Figure 2. Differences in weekly counts, date of report minus date of specimen. Left: isolates. Right: organism types. Both fluctuate around zero, with substantial variance.

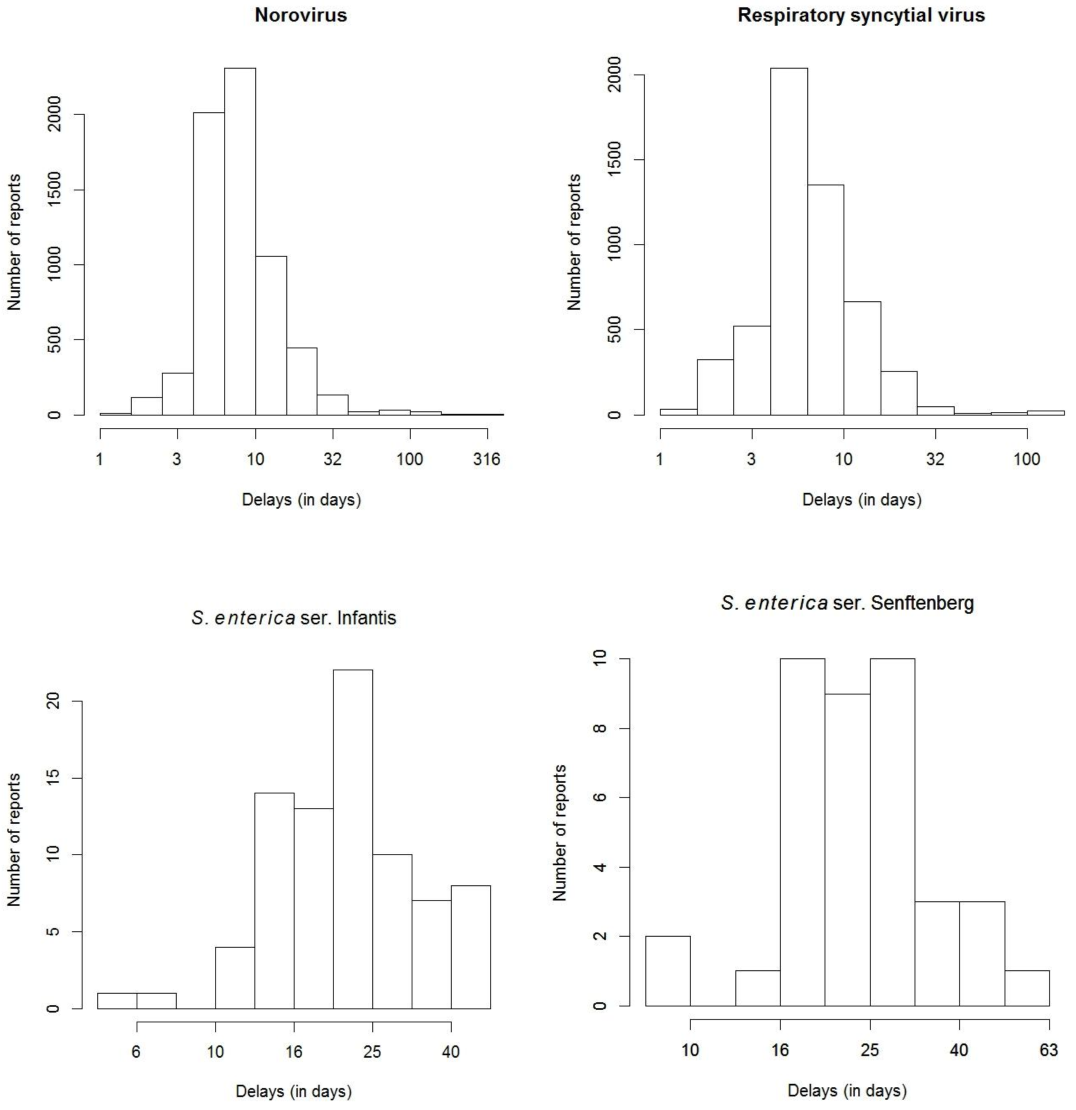

Technical Appendix Figure 3. Histograms of reporting delays (days) for four organisms. Modal delays are longer for the salmonellas, owing to the extra typing step involved.

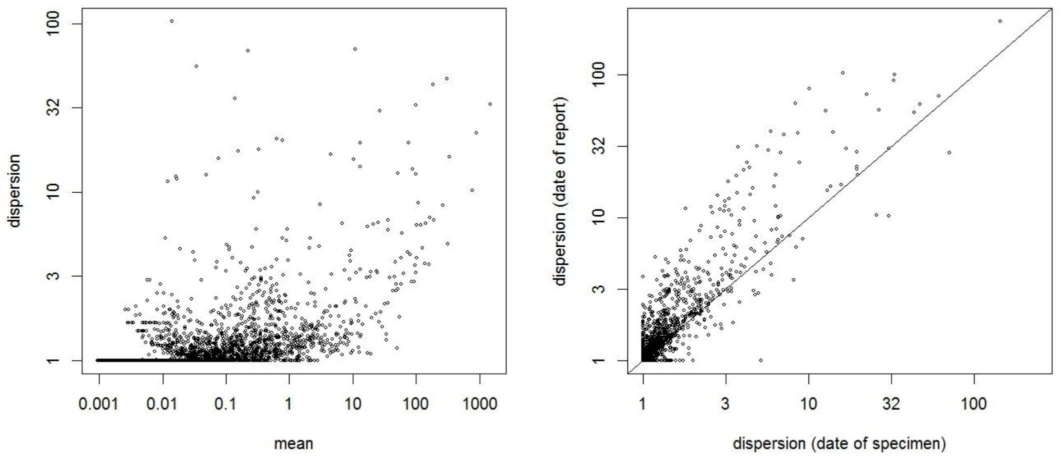

Technical Appendix Figure 4. Dispersion parameter. Left: plotted against mean count (on log scale). Right: for data by week of report and by week of specimen, with diagonal line.

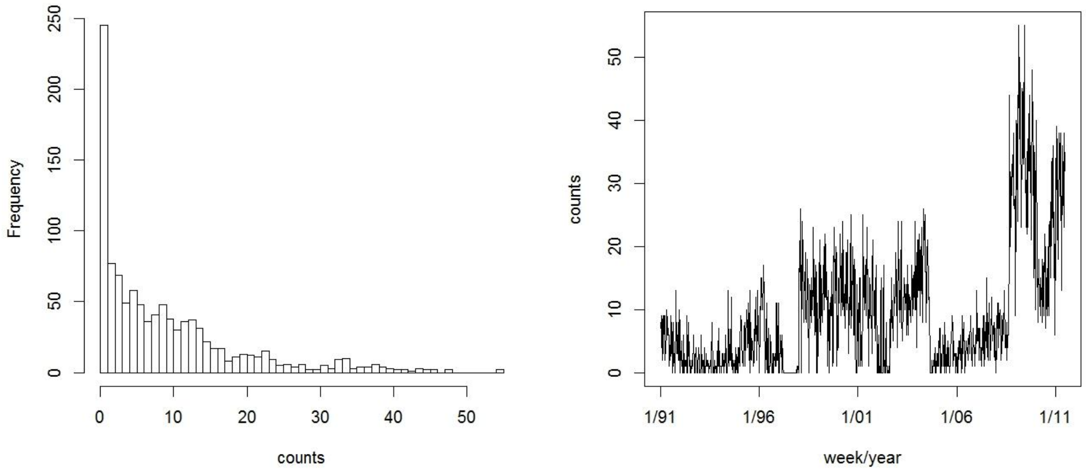

Technical Appendix Figure 5. Weekly counts of *Helicobacter pylori* isolates. Left: histogram, showing an excess of weeks with a count of zero. Right: time series of weekly counts, showing sudden changes in level, and a long run of zeroes between 1996 and 2001.
